# Supplementary material for: CRISPR-Cas12a targeting of ssDNA plays no detectable role in immunity
Source: Nucleic Acids Res. 2022 Jun 7;50(11):6414–22. doi: 10.1093/nar/gkac462 (PMC9226536; doi:10.1093/nar/gkac462)
Supplement: gkac462_Supplemental_File [file gkac462_supplemental_file.pdf]

Supplementary Materials

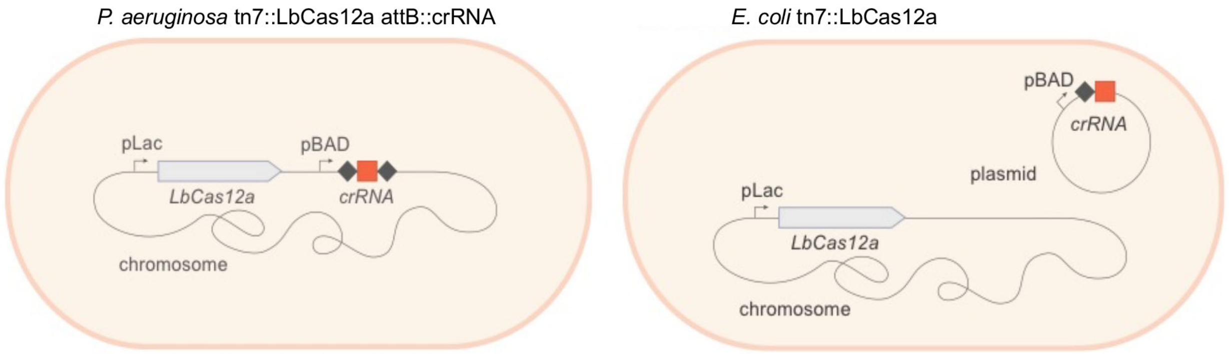

Figure S1. Schematic of the strains used in this study. *Pseudomonas aeruginosa* PAO1 strain was engineered to express chromosomally integrated LbCas12a and crRNA. *E. coli* BW25113 strain was engineered to express chromosomally integrated LbCas12a and plasmid-borne crRNA.

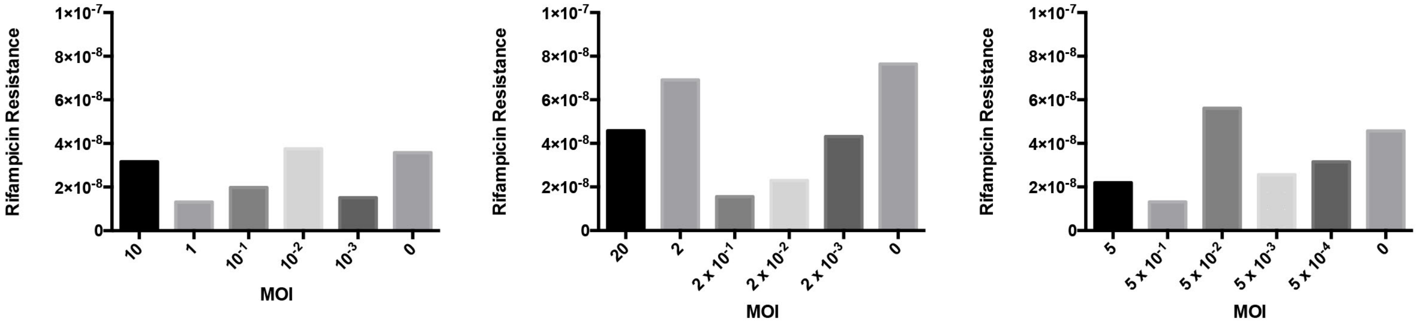

Figure S2. Rates of resistance to rifampicin after phage infection. Cultures of PAO1 expressing LbCas12a and crRNA were infected with varying multiplicities of infection (MOI) of DMS3 phage for 16 hours. For each experiment, surviving cells were plated in triplicate on LB agar with or without rifampicin to determine mutation frequency. Values indicate average number of rifampicin-resistant CFUs divided by total CFUs.

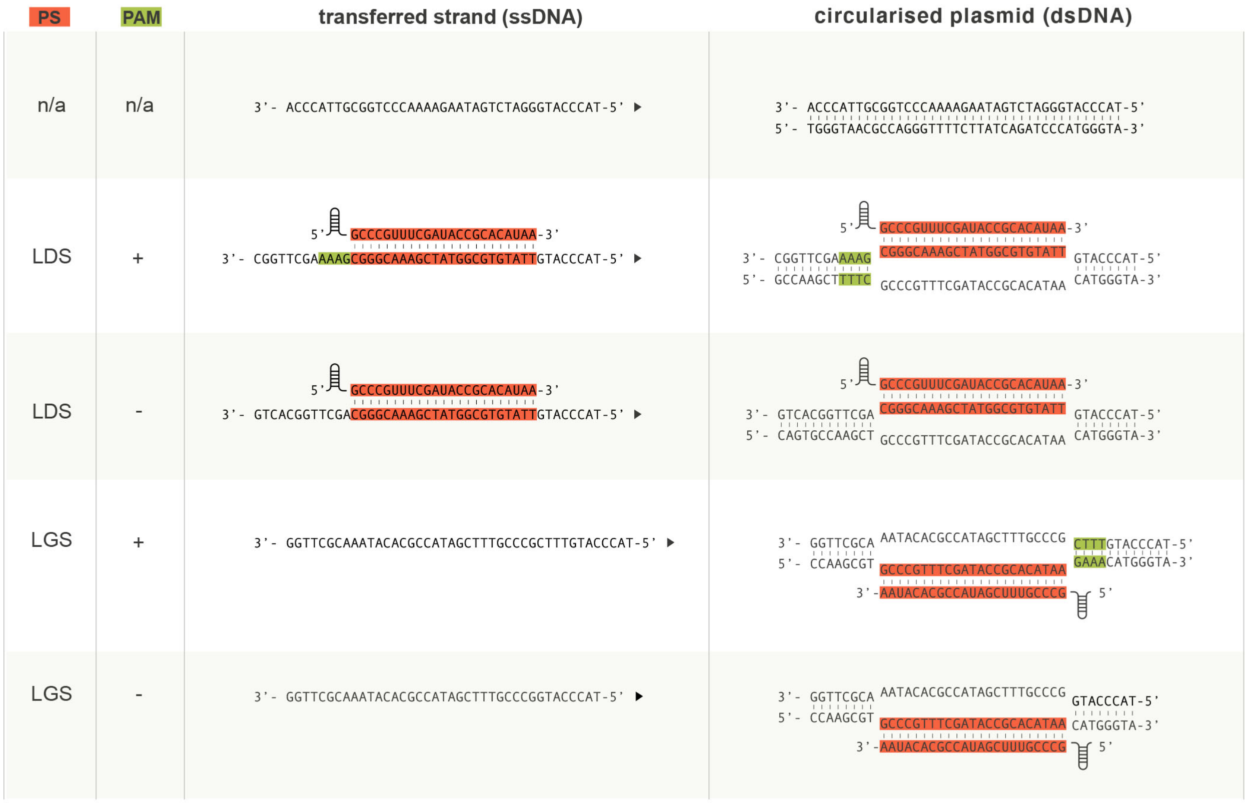

Figure S3. Schematic for crRNA and protospacers used for conjugation experiments. Five different pHERD30T-based plasmid constructs were used to assess for ssDNA *cis*-cleavage during conjugation. The spacer-protospacer matches are shown in red and the PAM sequence in green. The left panel depicts the presence or absence of complementary base-pairing between the crRNA and protospacers in the leading strand (transferred strand); the black arrow head indicates the direction of transfer during conjugation from the donor to the recipient cell. The right panel illustrates base pairing between the crRNA and protospacers in the plasmids once they become dsDNA. LDS, complementary protospacer is on the leading strand; LGS, complementary protospacer is on the lagging strand; PS, protospacer; PAM, protospacer adjacent motif.

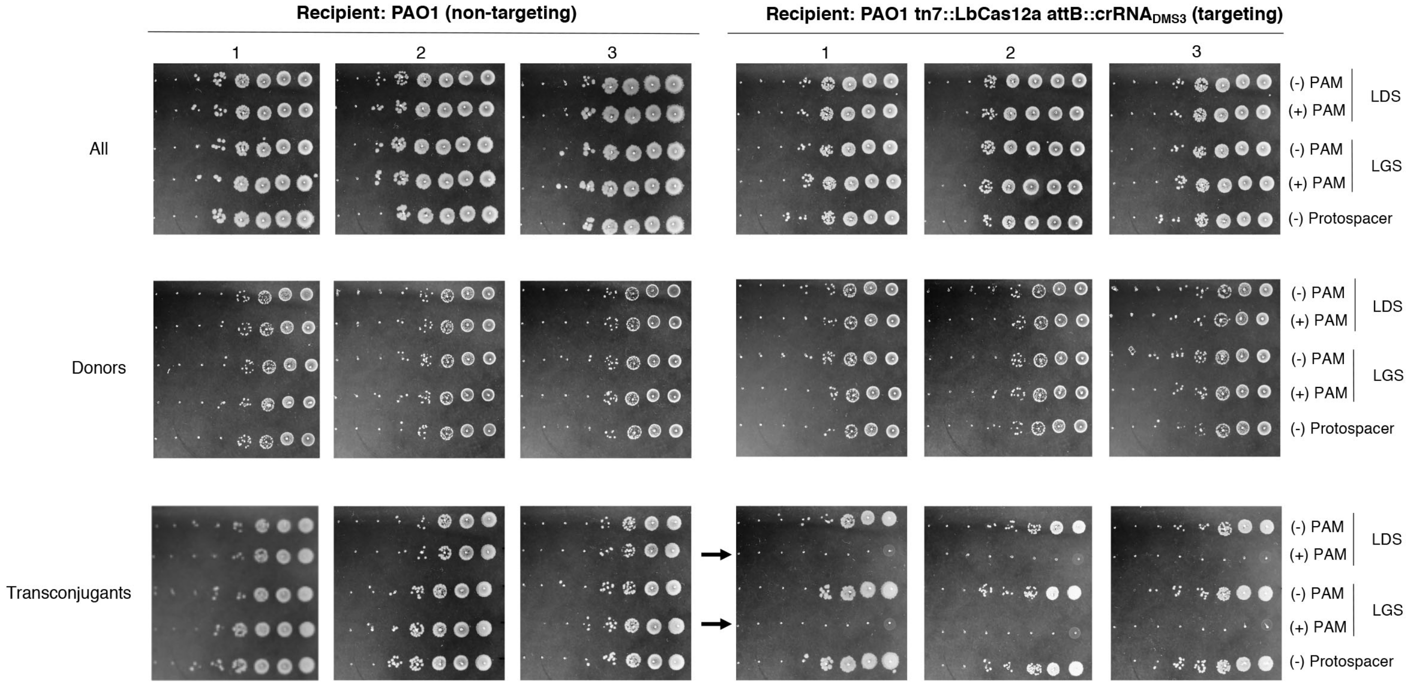

Figure S4. LbCas12a does not *cis*-cleave ssDNA during plasmid conjugation. Conjugation assays for ssDNA *cis*-cleavage. Colony forming units (10-fold dilution series) of transconjugants (bottom), donors (middle) and all (top) cells were grown in the appropriate selective media after the conjugative filter mating. Numbered columns indicate the three replicates for the combination of matings performed with either the non-targeting recipient (left) or the targeting recipient (right). Dilution rows start at 10^{-2} for "all" and 10^{0} for "donors" and "transconjugants". LDS, the complementary protospacer is on the leading strand; LGS, the complementary protospacer is on the lagging strand; (+/-) PAM indicates the presence/absence of the correct protospacer adjacent motif (PAM). Black arrows indicate where targeting is observed.

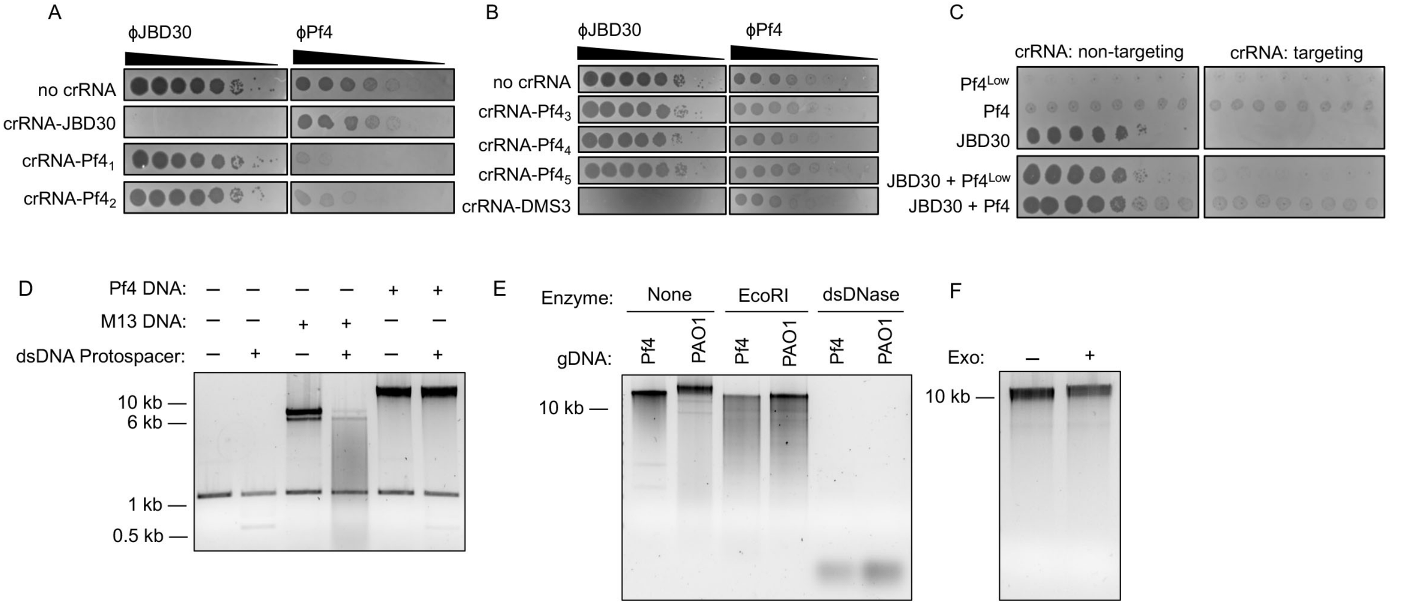

Figure S5. DNA purified from Pf4 virions is not susceptible to *trans*-cleavage. (A) Plaque assay for LbCas12a PAM-dependent cis-cleavage. Bacterial lawns of *P. aeruginosa* PAO1 strain were induced to co-express LbCas12a and crRNAs complementary to Pf4 or JBD30 protospacers. Upon plating, bacteria were singly infected with serial dilutions of JBD30 or Pf4 phage. Black spots indicate bacterial clearance due to phage replication. (B) Plaque assay for ssDNA cleavage by LbCas12a. Bacterial lawns of *P. aeruginosa* PAO1 strain

were induced to co-express LbCas12a and crRNAs complementary to Pf4 protospacers lacking a PAM. Upon plating, bacteria were singly infected with serial dilutions of JBD30 or Pf4 phage. crRNA complementary to a JBD30 protospacer with a PAM was used as a positive control for targeting. (C) Plaque assay for ssDNA trans-cleavage by LbCas12a. Bacterial lawns of *P. aeruginosa* PAO1 strain were induced to co-express LbCas12a and crRNA complementary to a JBD30 protospacer with a PAM. Upon plating, bacteria were singly infected or co-infected with JBD30 and Pf4 phage. Ten-fold serial dilutions of JBD were used while Pf4 was used at moderate and low titers. (D) *In vitro* LbCas12a cis- and trans-cleavage assay with dsDNA template (plus or minus protospacer) and DNA purified from Pf4 or M13 virions. (E) *In vitro* cleavage assay on DNA purified from PAO1 or Pf4 virions using enzymes as indicated. (F) *In vitro* cleavage assay on DNA purified from Pf4 virions using exonuclease I.

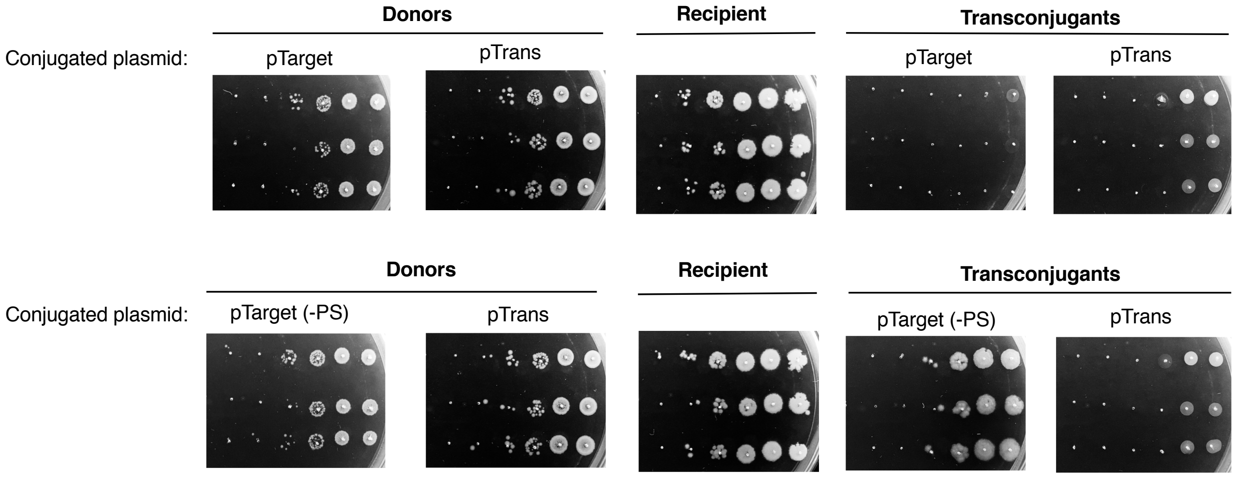

Figure S6. Conjugation assays for ssDNA *trans*-cleavage. The pictures show the colony forming units (10-fold dilution series) of donors (left), recipients (middle) and transconjugants (right) grown in the appropriate selection media after the co-conjugation filter mating experiment (triplicates in rows). The top panel shows the results for the co-conjugation of pTarget and pTrans. The bottom panel shows the results for the co-conjugation of pTarget(-PS) (which has the same sequence as pTarget but lacks the protospacer) and pTrans.

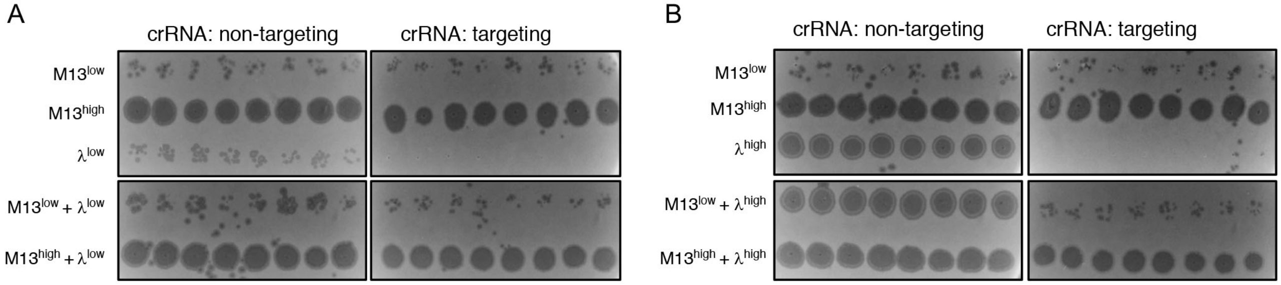

Figure S7. LbCas12a does not trans-cleavage M13 ssDNA during phage infection. Bacterial lawns of *E. coli* BW25113 F’ were induced to co-express LbCas12a and non-targeting or λ-targeting crRNA. Upon plating, bacteria were singly infected or co-infected with low or high titers of M13 and (A) low or (B) high titers of λ_{vir}.

Supplementary Table S1. Strains, phages, plasmids, and crRNAs used in this study.

The following bacterial strains, phages, plasmids, and crRNAs were used in this study. "Insert sequence" indicates the crRNA sequence encoded in the multiple cloning site of the specified plasmid to enable targeting of specified targets. The specified crRNA sequences were used for *in vitro* transcription and cleavage assays.

**Strains**	**Species**
PAO1	*Pseudomonas aeruginosa*
PAO1 tn7::LbCas12a	*Pseudomonas aeruginosa*
PAO1 tn7::LbCas12a attB::crRNA$_{DMS3}$	*Pseudomonas aeruginosa*
BW25113 F' tn7::LbCas12a	*Escherichia coli*
S17-1	*Escherichia coli*
CSH26	*Escherichia coli*

**Phages**
DMS3
JBD30
M13
λ_{vir}
Pf4

**Plasmids**		**Insert sequence**
pHERD30T **(pTarget(-PS))**		NA
pHERD20T		NA
pKJK5 **(pTrans)**		
Insert: protospacer		
pHERD30T-ps$_{DMS3}$-PAM **(pTarget)**		ttatgtgcggtatcgaaacgggc**gaaa**
pHERD30T-ps$_{DMS3}$		ttatgtgcggtatcgaaacgggc
pHERD30T-ps$_{DMS3}$-PAM-flip		**tttc**gcccgtttcgataccgcacataa
pHERD30T-ps$_{DMS3}$-flip		gcccgtttcgataccgcacataa
Insert: crRNA	**Target**	
pHERD30T-crRNA-DMS3	DMS3/JBD30	taatttctactaagtgtagatgcccgtttcgataccgcacataa
pHERD20T-crRNA-M13$_1$	M13	taatttctactaagtgtagatctgcaacagtgccacgctgagagc
pHERD20T-crRNA-M13$_2$	M13	taatttctactaagtgtagataacgaaccaccagcagaaga
pHERD20T-crRNA-λ	λ	taatttctactaagtgtagatgagggcagttgcggtcgtggaac
pHERD30T-crRNA-Pf4$_1$	Pf4	taatttctactaagtgtagatagacgttccggcgtcagcgcctg
pHERD30T-crRNA-Pf4$_2$	Pf4	taatttctactaagtgtagatgcaggctccagggagacagcgac
pHERD30T-crRNA-Pf4$_3$	Pf4	taatttctactaagtgtagatttttcatcatccacctcgcccat
pHERD30T-crRNA-Pf4$_4$	Pf4	taatttctactaagtgtagattcgggtcggaaggatcggtgttc
pHERD30T-crRNA-Pf4$_5$	Pf4	taatttctactaagtgtagatagaaggccagccccgccgcaatc
pHERD30T-crRNA-RFP	RFP	taatttctactaagtgtagatccgttacccaggactcctcc

**crRNA**	**Target**	**Sequence**

crRNA-DMS3	DMS3/JBD30	uaauuucuacuaaguguagaugcccguuucgauaccgcacauaa
crRNA-M13$_1$	M13	uaauuucuacuaaguguagaucugcaacagugccacgcugagagc
crRNA-M13$_2$	M13	uaauuucuacuaaguguagauaacgaaccaccagcagaaga

Supplementary Table S2. Features of LbCas12a targets used in this study.

The following sequences were used as Cas12a targets in this study. “Protospacer sequence” indicates sequence in the “target” mobile genetic element that corresponds to the indicated crRNA. PAM sequences are highlighted in bold where present. The gene name, accession number, and features of the gene targeted by the crRNA, as well as the strand on which the specified protospacer is encoded, are included where applicable.

**crRNA**	**Protospacer sequence**	**Target**	**Gene name**	**Accession**	**Strand**	**Features**
crRNA-DMS3	ttatgtgcggtatcgaaacgggc**gaaa**	DMS3	DMS3_21	YP_950445.1	template	structural protein
crRNA-DMS3	ttatgtgcggtatcgaaacgggc**gaaa**	JBD30	JBD30_024	AFQ21938.1	template	structural protein
crRNA-DMS3	**tttc**gcccgtttcgataccgcacataa	pTarget (flipped)			template	
crRNA-DMS3	ttatgtgcggtatcgaaacgggc**gaaa**	pTarget			coding	
crRNA-M13$_1$	ctgcaacagtgccacgctgagagc	M13	M13p10	927337	coding	phage assembly protein
crRNA-M13$_2$	aacgaaccaccagcagaaga	M13	M13p10	927337	coding	phage assembly protein
crRNA-λ	gagggcagttgcggtcgtggaac	λ		AAA96536.1	template	capsid component; 533
crRNA-Pf4$_1$	agacgttccggcgtcagcgcctg	Pf4	PA0728	AAG04117.1	coding	integrase
crRNA-Pf4$_2$	gcaggctccagggagacagcgac	Pf4	PA0728	AAG04117.1	coding	integrase
crRNA-Pf4$_3$	ttttcatcatccacctcgcccat	Pf4	PA0715	AAG04104.1	coding	reverse transcriptase
crRNA-Pf4$_4$	tcgggtcggaaggatcggtgttc	Pf4	PA0724	AAG04113.1	coding	coat protein
crRNA-Pf4$_5$	agaaggccagccccgccgcaatc	Pf4	PA0725	AAG04114.1	coding	hypothetical protein
crRNA-RFP	ccgttacccaggactcctcc	NA				
